# Loot

**Published:** 1870-11

**Authors:** 


### Loot.

Wm. H. Bradley, M.D., in the Pennsylvania Transactions, 1870, recommends in *Scarlatina* for the general and aphthous condition:

℞. Sodæ Sulphitis, - - - - - - - - - ʒ ss;

Sp. Ether. Nitr., - - - - - - - - - ʒ ij;

Syr. Gaultheriæ, - - - - - - - - - ʒ vj;

Aq., - - - - - - - - - - - ℥ ss;

M. S. A dram every three hours.

Conjoined with this, as an expectorant and anti-spasmodic:

℞. Syr. Prun. Virgin., - - - - - - - - - ℥i ;j

Syr. Scillæ, - - - - - - - - - - ℥ j;

Ext. Belladon. Fl., - - - - - - - - gtt. xxiv;

Ext. Verat. Virid. Fl., - - - - - - gtt. xxxvj;

Tr. Lobeliæ, - - - - - - - - - - ʒ ij;

M. S. A teaspoonful every three or four hours.

When the aphthous condition abated, in about a week, he prescribed as an alterative and tonic:

℞. Tinct. Ferri. Chlor., - - - - - - - - ʒj;

Potassæ Chlorat., - - - - - - - - - ʒ ij;

Syr. Gaultheriæ, - - - - - - - - - ℥ iij;

M. S. A dram three times a day.

The same gentleman observes (*op. citat.*, p. 99):

"The treatment I have adopted the past two years in fevers of the remittent, typhus, malarial, and typhoid types, has been with the sulphite of soda and quinine. At the outset, I usually give an alterative and cathartic dose, composed of—

| | | |
|---|---|---|
| ℞. Mass. Hydrarg., | - - - - - - - - | gr. x; |
| Ipecac. Pulv., | - - - - - - - - - - | gr. ss; |
| Quiniæ Sulph., | - - - - - - - - | gr. ij; |

M. Ft. mass in pil. iij.

To an adult at night, followed in the morning, or in six or eight hours, by a gentle aperient.

"I then prescribe the sulphite as follows:

| | | |
|---|---|---|
| ℞. Sodæ Sulphitis, | - - - - - - - - | ʒ ij; |
| Ipecac. Pulv., | - - - - - - - - - - | gr. vj; |
| Quiniæ Sulph., | - - - - - - - - | gr. xij; |

M. Divide into twelve powders; one powder every three hours.

"Conjointly with this, as a refrigerant diaphoretic, either of the following:

| | | |
|---|---|---|
| ℞. Liq Ammoniæ Acetatis, | - - - - - - | f ℥ vj; |
| Spts. Æther. Nit., | - - - - - - - - - - | f ℥ ss; |

M. f ℥ ss. every three hours.

Or—

| | | | | |
|---|---|---|---|---|
| ℞. Acid. Citric., - | ʒ iij; | ℞. | Potas. Carb., - | ʒ ij, ℈ij; |
| Aquæ, - - | f ℥ iv; | | Spts. Æther. Nit., | f ℥ ss; |
| | | | Aquæ, - - | f ℥ iijss; |

M. One tablespoonful every three hours in a state of effervescence alternately with the powders.

"In remittents, giving the powders during the remission every hour or two, and either of the above solutions, during the paroxysm of fever, I generally succeed in breaking up the paroxysm in a week or ten days. Since adopting the above treatment, I have not had a case of enteric fever continue over four weeks, and have in nearly every instance witnessed the controling influence of the remedy over the disease. In cases of great cerebral exaltation, I usually increase the amount of ipecac. in the powders, sufficient to produce slight nausea, and in great depression of the nervous centres, the amount of quinine, and if conjoined with symptoms of great debility, as is often the case, add strongly concentrated animal food, milk, beef essence, etc. In cases of great wakefulness, with delirium attendant upon adynamic conditions, as evinced in the widely dilated pupils and other correlative symptoms, I always give opiates either by the mouth, or hypodermically, either with the syringe or upon blistered surfaces. In cases of great tenderness in the iliac region, with great tympanitis, I prescribe sinapisms freely, and afterward cover the part with a broad flannel bandage, but I have seldom seen annoying tympanitis in this disease since using the sulphite of soda, and in only two instances the past year have I prescribed the ol. terebinth., the condition indicating its use not being developed in the other cases.

"The rationale of the above treatment of typhoid fever, so far as the sulphite of soda is concerned, I am not prepared to expatiate upon; but in my opinion (and I have experimented faithfully and impartially with the remedy), it seems to have a marked effect upon the 'materies morbi.' How it would act in epidemic typhoid, I have had no opportunity of testing, but my confidence in it as a remedial agent would induce me to give it an impartial trial should opportunity present."

Dr. John L. Atlee, of Lancaster (Pennsylvania Transactions, 1870,) gives this account of his experience with a criminal who had taken poison:

"Upon entering the room I found the patient in bed, lying upon his back, his countenance flushed, and with a stern and almost demoniac expression; his pulse natural, skin moist, and his intellectual faculties perfect. Dr. Baker informed me that he suspected poison, and had endeavored to administer emetics; but that the patient had obstinately refused all medical assistance. While receiving this information a violent spasm seized the patient, which I observed was of a decidedly tetanic character, similar to those previously observed, and which affected all the voluntary muscles. I told the doctor that I was satisfied the patient had taken strychnia. He had frequently declared before his trial, that, if convicted, he would never suffer imprisonment.

"Finding the patient obstinately bent on self-destruction, the use of the stomach-pump was suggested, and Dr. B. went to procure it. In his absence I used all my endeavors to induce the patient to take the emetic, without avail, and while pressing the cup to his lips, I discovered that his jaws were firmly closed, that he had a full set of teeth, and that it would be impossible, in a man of his muscular strength and determination, to use the stomach pump. It then occurred to me that by the use of chloroform a double object could be gained—a subjection of the patient, and a relief from the spasms. I went next door to the druggist, where I found Dr. B. preparing the stomach-pump, procured two ounces of chloroform, and with him returned to the patient, whom I had left in the care of a student.

"While at the druggist's, I ascertained that at the April term of the court, when he had expected the trial to come on, the patient had purchased there 20 grains of strychnia. This confirmed suspicion as to the cause of the symptoms, and showed the evident bent of his mind. On our return we found the spasms increased in force and frequency.

"By means of a folded napkin the chloroform was applied to the mouth and nostrils; but no sooner was it done than he grasped the napkin violently with his right hand, tore it away, and openly defied me to administer remedies to him. I then appealed to the gentlemen attending court, who by this time nearly filled the room, to assist us in holding down the patient, and by this means—having two persons to each arm and leg, and having the head firmly held, I was enabled to use the anæsthetic. In a few moments it produced its effect; his whole muscular system became relaxed; and he remained for ten or fifteen minutes perfectly quiescent. As soon as he was restored to consciousness, we found that a total change, mental, moral, and physical, had taken place. The spasms had left him, the expression of his countenance was calm and comparatively pleasant, and he was perfectly obedient to every request made of him. He took the emetic without difficulty, and we soon had the satisfaction of seeing his stomach thoroughly evacuated. He had partaken of a portion of his dinner before swallowing the strychnia, which probably prevented a more rapid absorption of it. At this time, about 3 o'clock P. M., I had to leave him, for three hours, under the care of Dr. B., with the understanding that he would re-administer the chloroform, should there be a recurrence of the spasms. At 6 o'clock P. M. I found the patient apparently well; there had been no spasms; his mind was calm, his temper placid, and he complained of nothing but weakness. He had a good night, and the next morning, although suffering from muscular debility, and some unsteadiness in his gait, he was brought before the court, and sentenced to confinement in the Eastern Penitentiary, where, a few weeks afterwards, he committed suicide, by hanging, in his cell."

C. Hasbrouck, M.D., (Transactions New Jersey, 1870,) after the interruption of the paroxysms in malarious fevers, relies upon the following as a tonic pill:

| | | |
|---|---|---|
| ℞. | Acid. Arseniosi, - - - - - - - - | gr. 1-20; |
| | Ferri Redact., - - - - - - - - - - | gr. j; |
| | Quiniæ Sulph., - - - - - - - - | gr. j; |
| | Mucil. Acac., - - - - - - - - | q. s.; |

M. S. One such to be taken two or three times a day, for some time.

The same gentleman commends, for the relief of the severe pain accompanying Herpes Zoster, and also for the purpose of hastening the cure, the application of collodion once or twice a day, so as to keep up its compressing action on the skin.

Dr. C. H. Voorhees (New Jersey Transactions, 1870,) prescribed, associated with appropriate hygienic measures, the following, with success in a stubborn case of epileptiform seizures:

℞. Potassii Iodid., - - - - - - - - ʒ j;

Potassii Bromid., - - - - - - - - - ℥ j;

Ammon. Bromid., - - - - - - - - ʒ ijss;

Potass. Bicarb., - - - - - - - - - ℈ ij;

Infus. Calumb., - - - - - - - - - ℥ vj;

M. S. A teaspoonful before each of the three meals, and three at bed-time.

Dr. A. P. Dutcher commends in the vomiting concurrent with the evacuation of a large cavity in phthisis:

℞. Acid. Carbolic, - - - - - - - - gr. iv;
Glycerin, Tinct. Cinnamomi, - - - āā ℥ j;

M. S. A teaspoonful every four hours.

Here are a couple of old formulæ whose uses are suggested by their combination.

J. G. Nichol gives this:

℞. Pulv. Opi., - - - - - - - - - - ℥ j;

Acid Hydrochlor., - - - - - - - ʒ j;

Aq. Destill., - - - - - - - - - - ℥ xix;

M. Shake daily and digest 14 days. Strain and filter.
Dose.—Twenty to forty drops.

℞. Gum Camphoræ, - - - - - - - - - ʒ iij;

Chloroform, - - - - - - - - - - - ʒ j;

M. Ft Solut.

Rub this up with the yolk of a fresh egg, and add water q. s. for four ounce mixture. Each teaspoonful contains 5½ grains of camphor and 2 minims of chloroform. When kept long it requires to be well shaken again.

***Malarial Poisoning.***

From the *American Practitioner* we extract the following sensible observations:

"We are aware that it is contrary to the received opinion on the subject, but our experience has led us irresistibly to the conclusion, that the intercurrent congestions, irritations, and even inflammations which occur in marsh fevers should not be regarded as contraindications to the use of quinia.

"There is a peculiar cachexia produced by malarial poisoning, which commonly presents itself in subjects who have had repeated attacks of malarial fever, but may occur in those who, while exposed to the poison, have escaped the development of any febrile phenomena. The most prominent symptom of this condition is a peculiar anæmia. The patient becomes pale, bloodless, with an icterode hue of the skin. Accompanying this condition, and often preceding it, is an enlargement of the spleen, which is sometimes tender on pressure, and almost always the seat of a vague, uneasy feeling, amounting, in some cases, to actual pain. In the advanced stages of this affection, œdema of the lower extremities nearly always appears, or there may be general anasarca, with ascites. In the treatment of this condition, removal from the malarial to a healthy locality is of the first importance. This, with chalybeate tonics, is usually sufficient for cure. Where, however, this cannot be done, iron, quinia and the regular and systematic exhibition of aperients are required. The following combination we have found most efficient in such cases:

℞. Sulphate of Iron, Sulphate of Quinia, - - āā ʒ j.
Sulph. of Strychnia, - - - - - - - - gr. j.
Socotr. Aloes, - - - - - - - - - - - ℈ j.

Mix. Make into thirty pills, one of which should be taken three times a day.

"We have seldom found it necessary to use any other remedy in malarial cachexia, whether attended or not by dropsical effusions. The iron and quinia seldom fail in these cases to reduce the enlarged spleen, in a few weeks, to its normal size. We have seldom found it necessary, in these cases, to resort to hydragogue-cathartics or diuretics. The iron and quinia, with aperients, seem to fulfill every indication of treatment in these cases. Citrate of iron and quinia has often yielded equally good results. The ferro-cyanuret of iron, which stands high in the estimation of some physicians in chronic malarial poisoning, is in our opinion inferior to either of the preceding. We have used in some obstinate cases, especially those attended by frequent relapses of intermittent fever, in alternation with the iron and quinia, a combination of quinia and arsenic, according to the following prescription, with very salutary effects:

℞. Quinia Sulph., - - - - - - - - - ʒ j.

Liq. Potassæ Arsenitis, - - - - - - ʒ iij.

Acid Sulph. aromat., - - - - - - - - ʒ j.

Tr. Rhei, Syrp. Zinziber, - - - - - - āā ℥ ij.

M. Signa. A teaspoonful three times a day after meals.

"We have purposely abstained from alluding to many of the long list of antiperiodics, so called, for the reason that in our hands a large number of them have proved themselves useless in the marsh fevers which are met with in practice, and because we have, in our opinion, a genuine specific for them in the sulphate of quinine."

***Iodide of Potassium in Syphilis.***

Concerning the use of iodide of potassium in syphilitic skin diseases, Dr. McCall Anderson lays down the following rules:

1. The longer the interval which has elapsed between the contraction of the syphilitic taint and the development of the eruption, the more likely it is to be of service.

2. If the patient is cachectic, it is, as a rule, to be preferred to mercury, except in recent cases of syphilis, when the mercurial vapor bath, or some such treatment, is more likely to prove successful.

3. The more extensive the tertiary eruption, the more certain it is to yield to iodide of potassium; although to this rule there are numerous exceptions.

4. If there is any tendency to syphilitic disease of the nostrils or neighboring parts, iodide of potassium should be withheld, or given with great caution, for, if it produces coryza, it is very apt to aggravate the morbid conditions of the parts.

5. It should be given in full doses.

In explanation of the last rule, Dr. Anderson states that he considers ten grains as the proper dose in the majority of instances, while sometimes as much as thirty or forty, thrice daily, may be requisite. As a typical prescription he gives:

| | |
|---|---|
| Ferri Ammonio-citratis, - - - - - - - | ʒ iij; |
| Potassii Iodidii, - - - - - - - - | ʒ i; |
| Syrupi Zinziberis, - - - - - - - - | ℥ vj; |
| Infus. Gentian Co., - - - - - - - | ℥ viij; |
| Aquæ, Ad., - - - - - - - - - - | ʒ xxjv; |

A tablespoonful in a large wineglassful of water thrice daily. —*Med. Gazette.*

***Ice in Aneurism.***

The *Medical Times and Gazette* records a case of successful treatment of aneurism of innominate artery, by application of ice bags. The patient was a man aged about 35. First noticed a swelling in the right side of the root of the neck, which increased very rapidly, so that in three weeks he was nearly suffocated by it, with excessive pain about the shoulder and behind the ears. The tumor was about the size of an orange, thin walled, with violent pulsations.

The man was at once put to bed and kept at perfect rest. A bladder of ice was applied over the aneurism, and this treatment has been kept up constantly since. For a while perfect rest was maintained, but free meat diet was permitted from the first, and the aneurism made marvellous progress towards recovery. There is no obvious prominence, and although one still detects a considerably extended area of impulse, yet this is quite quiet, and manipulation gives no pain.—*Ohio Reporter.*

***Arsenic Hypodermically.***

Dr. Lipp, of Graz, mentions six cases of psoriasis treated by hypodermic injections of arsenic acid. He has two solutions—one of three and the other of six grains of arsenic acid to one ounce of water, and injects from one-twenty-fifth to one-fifth of a grain of the acid daily. He had obtained successful results in three cases after having used respectively seven, three and one-half, and three grains of the acid. This method seems to present several advantages: in not deranging the digestive organs; in the smaller dose; and in the quicker cure. There occurred, in several cases, augmentation of thirst, with increased amount of urine, diminution of appetite, acceleration of the pulse, headache, vertigo, when the large doses were employed.—*Archiv. Dermat. und Syphilis.*

There has just been found at Paris a singular society that numbers already more than a hundred members. The members, by a formal clause in their wills, declare that they do not wish to be interred after their death; on the contrary, they direct that their bodies shall be given to the amphitheatres to be dissected. They form this resolution for the purpose of contributing as much as possible to the progress of that important science, without which a profound study of the art of healing is impossible. They expect, moreover, to help to do away with the prejudice against the dissection of bodies.—*Revue Therapeutique.*

Dr. Guyot gives from 30 to 60 grains of phosphate of lime in profuse perspiration, and especially in the night sweats incident upon phthisis pulmonalis. He mixes the phosphate with loaf sugar, and requires the patient to take a pinch of the powder frequently through the day.—*Ibid.*

***Protracted Gestation.***

Dr. L. L. Bedell reports (Leavenworth Medical Herald, August, 1870) having delivered a woman in her sixth pregnancy, June 12, 1870, 10 o'clock A. M., of a healthy female child, weighing nine pounds. After the delivery the mother informed him that her catamenia had ceased July 25, 1869, and that she had expected to be confined about the 1st of May, 1870; that in her previous gestations she had been able to calculate pretty accurately the period of her confinement. If this statement be correct, that her last menstruation ceased July 25, 1869, 10 months and 18 days elapsed before her delivery, or 322 days, and if we deduct 10 days after the disappearance of her menstrual flow, we will still have a period of gestation of 312 days.

**Removal of the Whole Larynx.**

In cases of malignant new growths in the larynx, which are beyond the possibility of extirpation, the question as to the removal of the whole larynx may be raised. This has recently been done by Dr. V. Czerny of Vienna, who has approached this question experimentally, and shows that in dogs the operation can be performed without great difficulty, and that the loss of the larynx is not necessarily fatal to them. Even if the epiglottis have been included in the removal, the dog can swallow his food. The respiration is carried on through a cannula. Dr. Czerny further experimented on the possibility of artificially supplying a dog in this condition with the means of phonation, and succeeded in this by adding to the upper part of the cannula a piece with metallic tongs. Every surgeon will agree with the conclusion of Dr. Czerny's interesting paper, that only the dreadful and hitherto hopeless state of patients with malignant laryngeal growths can justify the proposal of such an operation.—*Brit. Med. Jour.*

